# Rosmarinic acid is a novel inhibitor for Hepatitis B virus replication targeting viral epsilon RNA-polymerase interaction

**DOI:** 10.1371/journal.pone.0197664

**Published:** 2018-05-21

**Authors:** Yuta Tsukamoto, Sotaro Ikeda, Koji Uwai, Riho Taguchi, Kazuaki Chayama, Takemasa Sakaguchi, Ryo Narita, Wan-Ling Yao, Fumihiko Takeuchi, Yukie Otakaki, Koichi Watashi, Takaji Wakita, Hiroki Kato, Takashi Fujita

**Affiliations:** 1 Laboratory of Molecular Genetics, Department of Virus Research, Institute for Frontier Life and Medical Sciences, Kyoto University, Kyoto, Japan; 2 Institute of Molecular Medicine, University Hospital Bonn, University of Bonn, Bonn, Germany; 3 Laboratory of Molecular and Cellular Immunology, Graduate School of Biostudies, Kyoto University, Kyoto, Japan; 4 Division of Sustainable and Environmental Engineering, Graduate School of Engineering, Muroran Institute of Technology, Muroran, Japan; 5 Department of Gastroenterology and Metabolism, Applied Life Science, Institute of Biomedical & Health Science, Hiroshima University, Hiroshima, Japan; 6 Liver Research Project Center, Hiroshima University, Hiroshima, Japan; 7 Department of Virology, Graduate School of Biomedical Sciences, Hiroshima University, Hiroshima, Japan; 8 Centre for Structural Biology, Department of Molecular Biology and Genetics, Aarhus University, Aarhus, Denmark; 9 Department of Virology II, National Institute of Infectious Diseases, Tokyo, Japan; 10 Department of Applied Biological Science, Tokyo University of Science, Noda, Japan; 11 CREST, Japan Science and Technology Agency (JST), Saitama, Japan; Academia Sinica, TAIWAN

## Abstract

Current therapeutics for hepatitis B virus (HBV) patients such as nucleoside analogs (NAs) are effective; however, new antiviral drugs against HBV are still desired. Since the interaction between the epsilon (ε) sequence of HBV pregenomic RNA and viral polymerase (Pol) is a key step in the HBV replication cycle, we aimed to identify small compounds for its inhibition, and established a pull-down assay system for the detection of ε-RNA-binding-Pol. Screening showed that 5 out of 3,965 compounds inhibited ε-Pol binding, and we identified rosmarinic acid, which exhibited specificity, as a potential antiviral agent. In order to examine the anti-HBV effects of rosmarinic acid, HBV-infected primary human hepatocytes from a humanized mouse liver were treated with rosmarinic acid. The rosmarinic acid treatment decreased HBV components including the amounts of extracellular HBV DNA with negligible cytotoxicity. We also investigated the combined effects of rosmarinic acid and the NA, lamivudine. rosmarinic acid slightly enhanced the anti-HBV activity of lamivudine, suggesting that the HBV replication step targeted by rosmarinic acid is distinct from that of NA. We analyzed an additional 25 rosmarinic acid derivatives, and found that 5 also inhibited ε-Pol. Structural comparisons between these derivatives implied that the “two phenolic hydroxyl groups at both ends” and the “caffeic acid-like structure” of rosmarinic acid are critical for the inhibition of ε-Pol binding. Collectively, our results demonstrate that rosmarinic acid inhibits HBV replication in HBV-infected cells by specifically targeting ε-Pol binding.

## Introduction

Hepatitis B virus (HBV) infection is a major health issue worldwide, with approximately 248 million chronically infected individuals (CHB) [1]. Approximately 686,000 HBV-related deaths occur annually [2]. Interferon-α (IFN-α), pegylated IFN-α (PEG-IFN-α), and six nucleos(t)ide analogues (NAs), including lamivudine, entecavir, adefovir dipivoxil, tenofovir disoproxil fumarate, tenofovir alafenamide, and telbivudine, are currently approved for use in the clinical treatment of CHB patients [3]. Treatments with IFN have the potential to achieve HBsAg seroclearance by immunomodulation; however, not all patients respond to IFN. Although NAs more strongly suppress HBV replication than IFN by inhibiting reverse transcription (RT) with less side effects, the discontinuation of NAs may result in the relapse of HBV. Thus, life-long treatments with NAs are required, but may result in the emergence of resistant virus variants [4]. Since current therapeutics for CHB are insufficient, novel anti-HBV drugs are urgently required.

cccDNA serves as a template for all transcripts of HBV; therefore, it represents an attractive target for chronic HBV infection. Studies on zinc-finger nucleases (ZFNs), transcription activator-like effector nucleases (TALENs), and RNA-guided clustered regulatory interspaced short palindromic repeats (CRISPR)-Cas endonucleases were performed in order to specifically eliminate hepadnaviral cccDNA [5–10]. The small compounds, CCC-0975 and CCC-0346, were identified as inhibitors of the conversion from rcDNA to cccDNA [11]. However, their use as a clinical trial approach is difficult because the strategy of targeting cccDNA is associated with the serious risk of side effects due to off-targeting. Although many types of inhibitors targeting different replication steps, including AT-61 and AT-130 (pregenomic RNA (pgRNA) encapsidation), Bay 41–4109 (capsid formation), nucleic acid polymers (multistep including secretion), and Myrcludex-B (entry), have been identified [12–17], a novel drug has not yet been applied to clinical anti-HBV therapy.

HBV Pol functions in many essential steps of HBV replication including RT, DNA synthesis, and RNA degradation. In addition to RT, the RNase H activity of Pol is a possible therapeutic target. Tavis and colleagues identified specific inhibitors for the RNase H activity of HBV Pol [18–21]. Besides its enzymatic roles, HBV Pol is crucially involved in pgRNA encapsidation. HBV Pol interacts with the ε sequence of pgRNA, and the ε-Pol interaction is an indispensable step for encapsidation [22]. Previous studies revealed that porphyrin compounds including hemin suppressed the ε-Pol interaction and subsequent protein-priming reaction [23]. Carbonyl J acid derivatives, known as HIV-1 RT inhibitors, have also been identified as ε-Pol binding and protein-priming inhibitors [24].

Since the interaction between ε and Pol is a distinct step from RT, ε-Pol binding inhibitors may be promising agents for combination therapy with NAs. However, large-scale screening to identify ε-Pol binding inhibitors has not yet been conducted. We herein established a screening system to search for ε-Pol binding inhibitors, and performed it using three chemical libraries including United States Food and Drug Administration (FDA)-, European Medicines Agency (EMA)-, and other agency-approved compounds. As a result of screening, rosmarinic acid and quercetin were identified as novel and specific inhibitors of ε-Pol binding. Quercetin has been reported to inhibit HBV replication. Therefore, we analyzed the anti-HBV effects of rosmarinic acid. Furthermore, an analysis of several types of rosmarinic acid derivatives revealed the critical structural features of rosmarinic acid for the inhibition of ε-Pol binding.

## Materials and methods

### Cell culture and reagents

HEK-293T [25] and HepG2 [26] cells were maintained in Dulbecco’s modified Eagle’s medium (DMEM) (Nacalai Tesque, 08459) containing 10% fetal bovine serum (FBS), 100 U/ml penicillin, and 100 μg/ml streptomycin. Hep38.7-Tet cells [27] were maintained in DMEM containing 5% FBS, 100 U/ml penicillin, 100 μg/ml streptomycin, 400 μg/ml G418, and 400 ng/ml tetracycline. PXB-cells were purchased from PhoenixBio (PPC-P01) and cultured in dHCGM (DMEM containing 10% FBS, 100 U/ml penicillin, 100 μg/ml streptomycin, 20 mM HEPES, 44 mM NaHCO_3_, 15 μg/ml L-proline, 250 ng/ml human recombinant insulin, 50 nM dexamethazone, 5 ng/ml human recombinant epidermal growth factor (EGF), 100 μM ascorbic acid, and 2% DMSO).

### Plasmids

p3×Flag CMV/pol (YE), an expression plasmid of 3×FLAG-Pol, was generated by Dr. Sakaguchi and Dr. Chayama (Hiroshima University, Japan). p3×FLAG-ISG20 was constructed as described below. p3×Flag CMV/pol (YE) was digested using HindIII and EcoRI to remove the cDNA of Pol, and subcloned ISG20 cDNA was then inserted into the cleaved vector. p3×FLAG-ISG20 D94G was generated using the KOD -plus- Mutagenesis Kit (TOYOBO, SMK101).

### Nucleic acids

ε-biotin RNA, ε RNA, control-biotin RNA, and DNA-biotin were purchased from Japan Bio Services. The sequence of ε RNA was 5'-ucucaUGUUCAUGUCCUACUGUUCAAGCCUCCAAGCUGUGCCUUGGGUGGCUUUGGGGCAUGGACAuugac-3', and that of control RNA was 5'-ucucaUGACGAGGCUGGGUGCUCCUUCUACGUGUUUUCGUUGUAGGUAGGACCCAAAUUGCCGCUCuugac-3' (Length and composition are the same as ε, whereas the sequence is different [randomized]). The 5' terminal “ucuca” and 3' terminal “uugac” were 2' O-Methyl RNA (for stabilization). ε-biotin and control-biotin were biotinylated at the 3' terminus. The sequence of DNA-biotin was 5'-TCTCATGTTCATGTCCTACTGTTCAAGCCTCCAAGCTGTGCCTTGGGTGGCTTTGGGGCATGGACATTGAC-3', and was biotinylated at the 3' terminus.

### Chemical libraries and compounds

The Tocriscreen compound library (Tocris Bioscience), Pharmakon1600 drug library (Microsource Discovery Systems), and Prestwick chemical library (Prestwick chemical) were used for screening. The Pharmakon1600 drug library was generously provided by Dr. Koh Takeuchi (Advanced Industrial Science and Technology, Ibaraki, Japan). The Prestwick chemical library was kindly gifted by Dr. Masayuki Shimojima and Dr. Masayuki Saijo (National Institute of Infectious Diseases, Tokyo, Japan). Rosmarinic acid, quercetin, merbromin, hemin, calcomine orange 2RS, and salvianolic acid A were purchased from Sigma-Aldrich (536954, Q4951, M7011, 51280-1G, C9519, and SML0045, respectively). Erythrosine B, and lamivudine were purchased from Tokyo Chemical Industry (T0557 and L0217, respectively). Verteporfin was purchased from Cayman Chemical (17334). Rosmarinic acid derivatives were synthesized as previously described [28].

### Compound treatment to cell lines stably expressing HBV

Hep38.7-Tet cells were seeded on collagen-coated 96-well plates at 1.0×10^4^ cells/well. After cell attachment (2 to 3 h), culture medium was replaced with fresh FBS-free medium containing compounds 0, 1, 2, 3 days after seeding. FBS was added to culture medium 2 to 3 h after it had been changed. On day 5, extracellular HBV DNA was extracted from the culture supernatant and quantified by qPCR, as described below, and cells were then subjected to the WST-1 cell proliferation assay (Takara, MK400) following the manufacturer’s instructions. CC50 in HepG2 cells was calculated from results at least 7 kinds of concentration of compound (Table 1).

**Table 1:** Citotoxicity of the compounds.

Compound	CC_50_ in HepG2 (μM)
Rosmarinic acid	116±34
Quercetin	>500
Erythrosine B	115±42
Merbromin	156±43
Verteporfin	1.11±0.35
Hemin	111±23
Salvianolic acid A	86±4
Compound 2	258±14
Compound 12c	239±89
Compound 13c	229±10
Compound 21a	356±105

### Compound treatment to primary human hepatocytes infected with HBV

PXB-cells were infected at 5 Geq/cell using dHCGM containing serum from HBV-infected chimeric mice with humanized livers (PhoenixBio, PPC-BC), and 4% polyethylene glycol 8,000. Culture medium was replaced with fresh dHCGM containing compounds on days 1, 2, and 7 post-infection. On days 7 or 12, extracellular HBV DNA was extracted from the culture supernatant and quantified by qPCR, HBV RNA was extracted from cells and quantified by RT-qPCR, and SHBs were measured by ELISA as described below.

### Quantitative real-time PCR (qPCR)

Extracellular HBV DNA was extracted using the SMITEST EX-R+D KIT (Medical and Biological Laboratories, GSJ0201) or Geno Plus Genomic DNA Extraction Miniprep System (Viogene, GG2002). HBV DNA levels were monitored with the StepOnePlus Real Time PCR System using the Fast SYBR PCR Master Mix (Applied Biosystems—Thermo Fisher Scientific), and the following primer set: qPCR primers for HBV DNA (Forward 5'-TTCACCTCACCATACAGCACTC-3', Reverse 5'- ATAGGGGCATTTGGTGGTCTG-3'). The copy number/μl of HBV DNA was measured using the HBV DNA fragment, which was amplified by the following primers: PCR primers for HBV DNA fragment amplification (Forward 5'-TTCACCTCACCATACAGCACTC-3', Reverse 5'- ATAGGGGCATTTGGTGGTCTG-3').

### Quantitative real-time reverse transcription PCR (qRT-PCR)

Total RNA was isolated using TRIzol reagent (Invitrogen—Thermo Fisher Scientific, 15596018) with DNase I (Roche, 4716728), and subjected to RT using a High-Capacity cDNA Reverse Transcription Kit (Applied Biosystems—Thermo Fisher Scientific, 4368813). mRNA levels were quantified by the StepOnePlus Real Time PCR System using Fast SYBR PCR Master Mix, and the following primer set: qRT-PCR primers for HBV RNA (Forward 5'-TTCACCTCACCATACAGCACTC-3', Reverse 5'- ATAGGGGCATTTGGTGGTCTG-3') and GAPDH (Forward 5'- ACTGCCAACGTGTCAGTGGT-3', Reverse 5'- TTACTCCTTGGAGGCCATGT-3').

### Enzyme-linked immunosorbent assay (ELISA)

Culture supernatants were analyzed using the HBs S Antigen Quantitative ELISA Kit, Rapid-II (Beacle, Inc., BC013).

### Electrophoresis mobility shift assay (EMSA)

EMSA was performed as described previously [29]. Briefly, a total of 30 pmol of the recombinant RIG-I protein was mixed with 5 pmol of synthetic dsRNA (25/25c) [29] and in a reaction mixture (20 mM Tris-HCl (pH 8.0), 1.5 mM MgCl_2_, and 1.5 mM DTT) in the presence of compounds. After an incubation at room temperature for 15 min, the reaction mixture was applied to a 15% acrylamide gel (TBE buffer) and dsRNA was detected by EtBr staining and RIG-I by Coomassie Brilliant Blue staining.

### Helicase assay

The RIG-I helicase assay was performed as described previously [29].

### Pull-down assay

HEK-293T cells were transfected with p3×Flag CMV/pol (YE) or p3×FLAG-ISG20 D94G. At 48 h post-transfection, cells were harvested and stored at -80°C. The cell pellet was lysed in lysis buffer (50 mM Tris-HCl (pH 8.0), 150 mM NaCl, 1% NP-40, 1 mM DTT, 2 μg/ml leupeptin, 1 mM PMSF, 1 mM vanadate, and 20 units of an RNase inhibitor) and centrifuged. The cleared lysate was incubated in the presence of compounds on ice for 20 min, mixed with RNAs (ε-biotin, ε, or control-biotin) and equilibrated with streptavidin sepharose (GE Healthcare, 17511301, GE Healthcare, Buckinghamshire, UK), incubated at 4°C for 2 h with rotation, collected by centrifugation, washed three times using lysis buffer, resuspended in SDS sample buffer (125 mM Tris-HCl (pH 6.8), 4% SDS, 20% glycerol, 0.01% BPB, and 10% 2-mercaptoethanol), incubated at 95°C for 3 min, and then analyzed by SDS-PAGE using a 7.5% acrylamide gel and immunoblotting using an anti-FLAG-Peroxidase (HRP) antibody (Sigma-Aldrich, A8592). The biotinylated DNA pull-down assay was performed as follows. One microgram of DNA-biotin was incubated in the presence of compounds in buffer (50 mM Tris-HCl (pH 8.0), 150 mM NaCl, and 1% NP-40), equilibrated with streptavidin sepharose at 4°C for 2 h with rotation, collected by centrifugation, washed three times, and DNA was recovered by extraction with TRIzol reagent. Recovered DNA was analyzed by 15% acrylamide gel electrophoresis, followed by staining with EtBr.

## Results

### Screening of ε-Pol inhibitors by a pull-down assay

**Fig 1:**
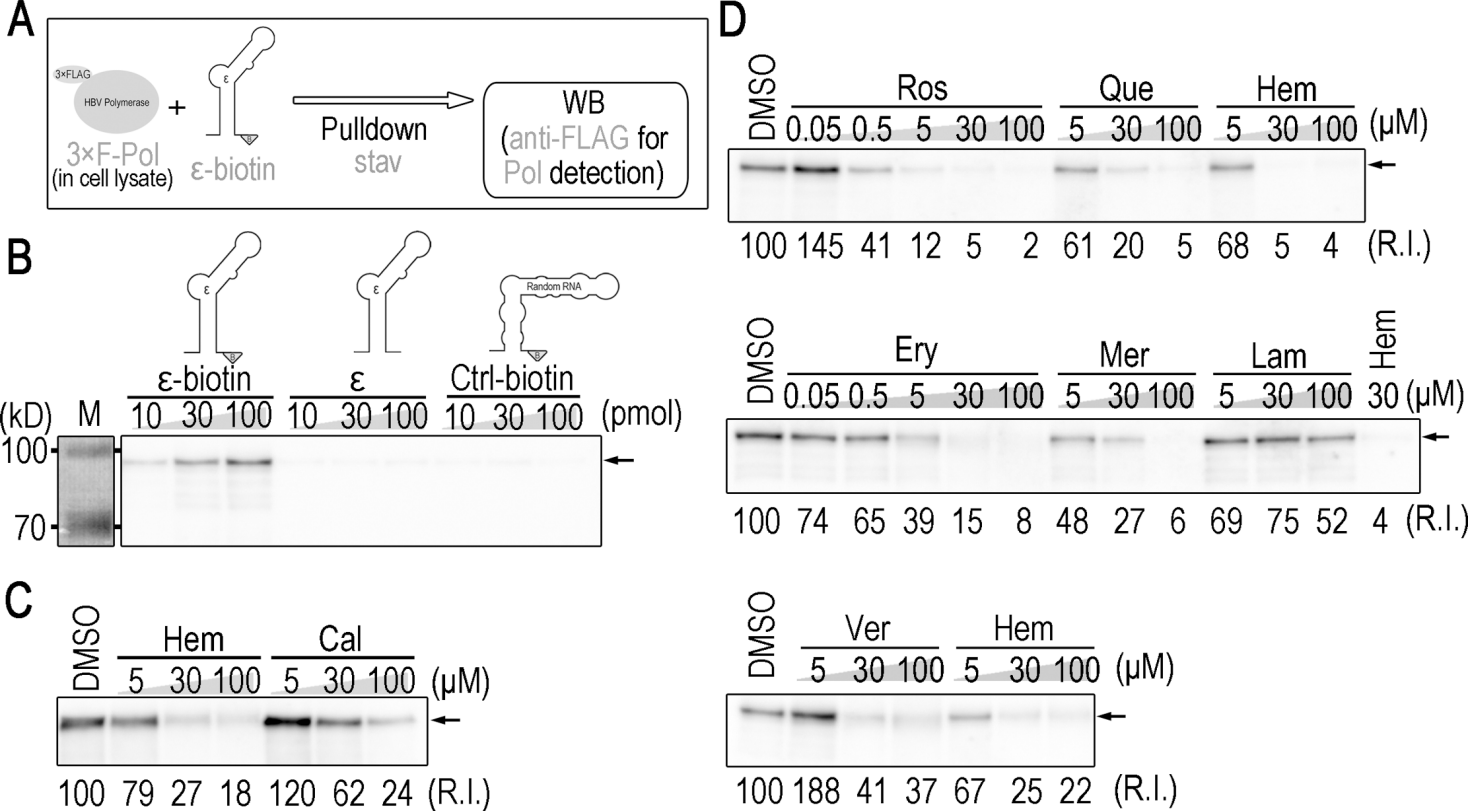
Five ε-Pol binding inhibitors selected by pull-down assay-based screening. (A) Scheme of the detection of ε-Pol binding by pull-down assays. The lysate of HEK-293T cells expressing 3×FLAG-Pol was incubated with ε-biotin, and pulled down using streptavidin sepharose. Precipitated 3×FLAG-Pol was detected by a Western blot analysis using an anti-FLAG antibody. (B) A Western blot analysis for Pol pulled-down by the indicated RNAs (ε RNA with biotin, ε RNA without biotin, and control RNA with biotin). (C and D) A Western blot analysis for Pol pulled-down by 10 pmol ε-biotin in the presence of the indicated compounds. Lamivudine was used as a negative control. Arrows: bands detected at a position of the estimated mass of full-length 3×FLAG-Pol.

In order to identify novel inhibitors of ε-Pol binding, we established an *in vitro* screening system based on a pull-down assay, as shown in Fig 1A. Briefly, chemically synthesized ε-biotin RNA was mixed with chemicals in the lysates of human cell lines expressing 3×FLAG-tagged-Pol, pulled down by streptavidin beads, and the ε-binding of 3×FLAG-Pol was then detected by Western blotting. As expected, 3×FLAG-Pol was pulled down with ε-biotin, but not with non-biotinylated ε or biotinylated RNA with an unrelated sequence (Fig 1B). Conditions were optimized further by using the previously reported inhibitors, hemin and calcomine orange 2RS [23, 24] (Fig 1C), and we screened for novel inhibitors using chemical libraries.

Primary screening was performed using compounds in a 10-drug mix, and candidate inhibitors were eventually identified by secondary screening using a single compound in the candidate 10-drug mix (S1A and S1B Fig). As a result, 5 out of 3,965 compounds were identified as inhibitors of ε-Pol binding. All 5 compounds: rosmarinic acid, quercetin, erythrosine B, merbromin, and verteporfin, exhibited inhibitory activities in dose-dependent manners (Fig 1D). Of note, verteporfin is a porphyrin, similar to hemin.

### Rosmarinic acid and quercetin are specific inhibitors of ε-Pol

**Fig 2:**
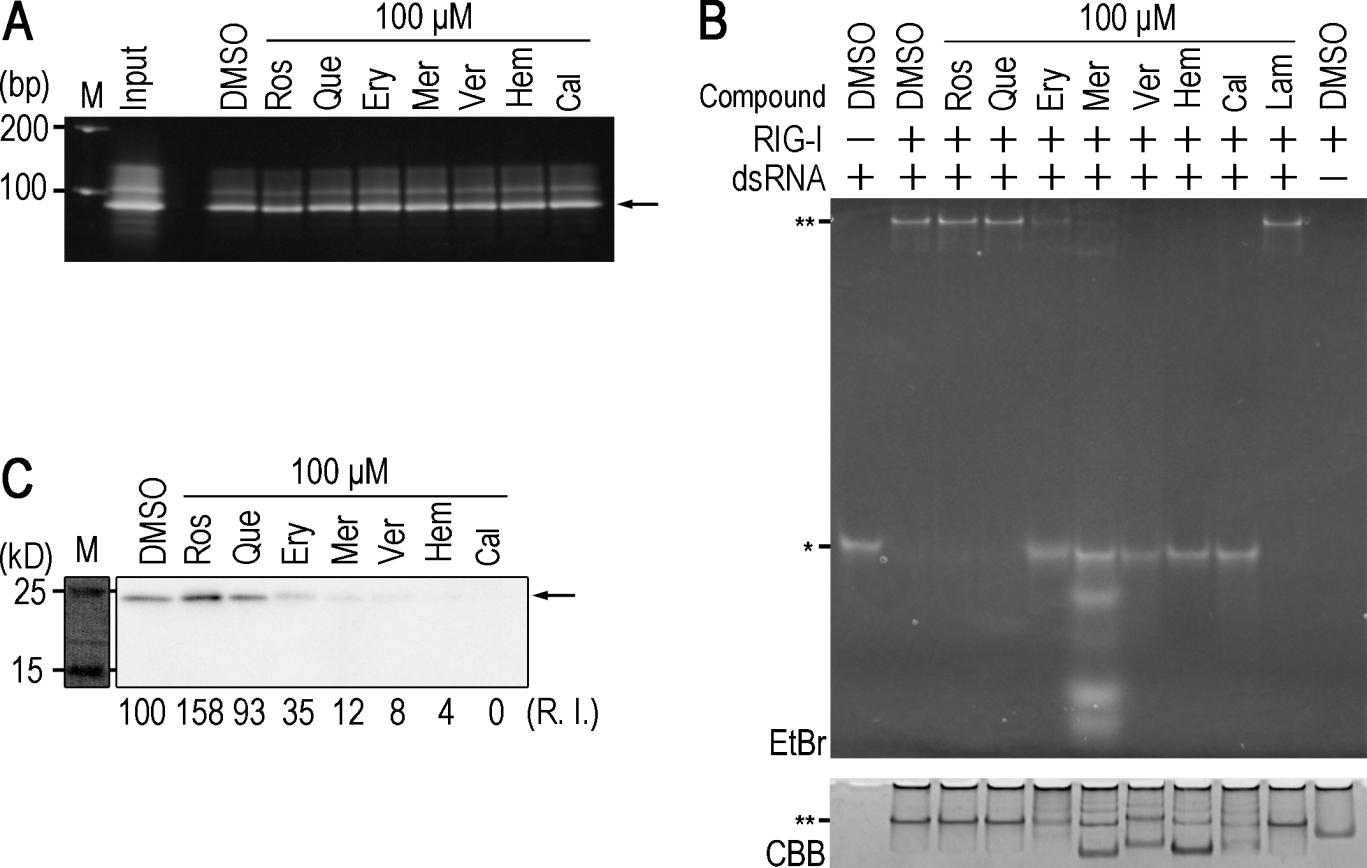
Rosmarinic acid and quercetin are specific inhibitors of ε-Pol binding. (A) Seventy-one-base ssDNA-biotin was pulled-down in the presence of the indicated compounds, and detected by EtBr staining. Arrow: 71-base ssDNA. (B) dsRNA-RIG-I EMSA in the presence of the indicated compounds. *: monomeric dsRNA, **: dsRNA-RIG-I complex. (C) A Western blot analysis for ISG20 D94G pulled-down by ε-biotin in the presence of the indicated compounds. Arrow: 3×ISG20 D94G.

We examined the specificities of the 5 compounds for the inhibition of ε-Pol. In order to exclude the possibility that these compounds inhibit biotin-avidin binding, biotinylated DNA was pulled down by streptavidin sepharose in the presence or absence of the compounds. None of the 5 compounds affected binding between DNA-biotin and streptavidin sepharose (Fig 2A), suggesting that these compounds target the protein-RNA interaction. A previous study reported that RIG-I recognizes dsRNAs, such as the ε sequence in HBV pgRNA, and subsequently exhibits anti-HBV activity [30]. In order to examine whether the compounds identified affect RIG-I-dsRNA binding activity, we conducted an electrophoresis mobility shift assay (EMSA) to investigate the interaction between dsRNA and RIG-I in the presence or absence of the compounds. Rosmarinic acid and quercetin did not affect dsRNA-RIG-I binding, whereas the other compounds, erythrosin B, merbromin, and verteporfin, as well as the previously reported chemicals, hemin and calcomine orange 2RS, inhibited dsRNA-RIG-I binding (Fig 2B). We also tested whether the 5 compounds affect RIG-I helicase enzymatic activity, and found that only rosmarinic acid and quercetin did not affect the helicase activity of RIG-I, suggesting that RIG-I signaling is not influenced by treatments with rosmarinic acid and quercetin (S2A Fig). In order to further validate specificity, another RNP complex was tested. Liu and colleagues recently reported that ISG20 interacted with ε in an enzymatic activity (exonuclease)-independent manner [31]. 3×FLAG-tagged ISG20 D94G, an exonuclease inactive mutant, was pulled down with ε-biotin in the presence or absence of the compounds. Rosmarinic acid and quercetin did not affect ε-ISG20 binding, whereas erythrosin B, merbromin, verteporfin, hemin, and calcomine Orange 2RS exerted inhibitory effects (Fig 2C). These results suggest that rosmarinic acid and quercetin specifically inhibit the ε-Pol interaction.

### Rosmarinic acid suppresses HBV replication

**Fig 3:**
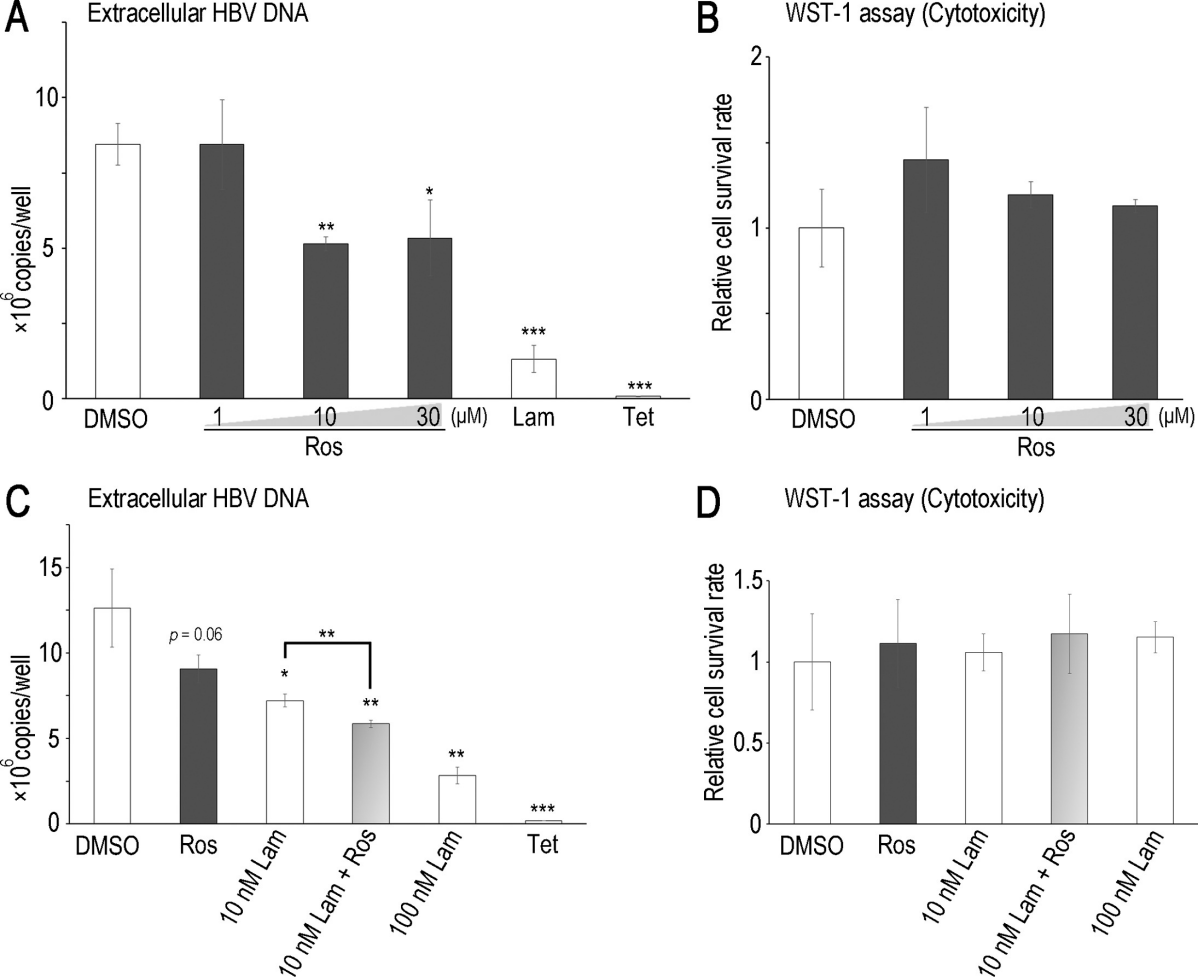
Rosmarinic acid inhibits HBV DNA production in cell lines stably expressing HBV. Hep38.7-Tet cells were treated with rosmarinic acid at the indicated concentrations, 100 nM lamivudine, or 400 ng/ml tetracycline (A and B), or with 30 μM rosmarinic acid and/or 10 nM lamivudine, 100 nM Lam, or 400 ng/ml tetracycline (C and D). Five days after the induction of HBV expression, extracellular HBV DNA was quantified by qPCR (A and C), and cell viability was measured using WST-1 reagent (B and D). Data are from one representative of at least two independent experiments; the means and S.D. of triplicate experiments are shown (*: *p* < 0.05, **: *p* < 0.01, ***: *p* < 0.001).

**Fig 4:**
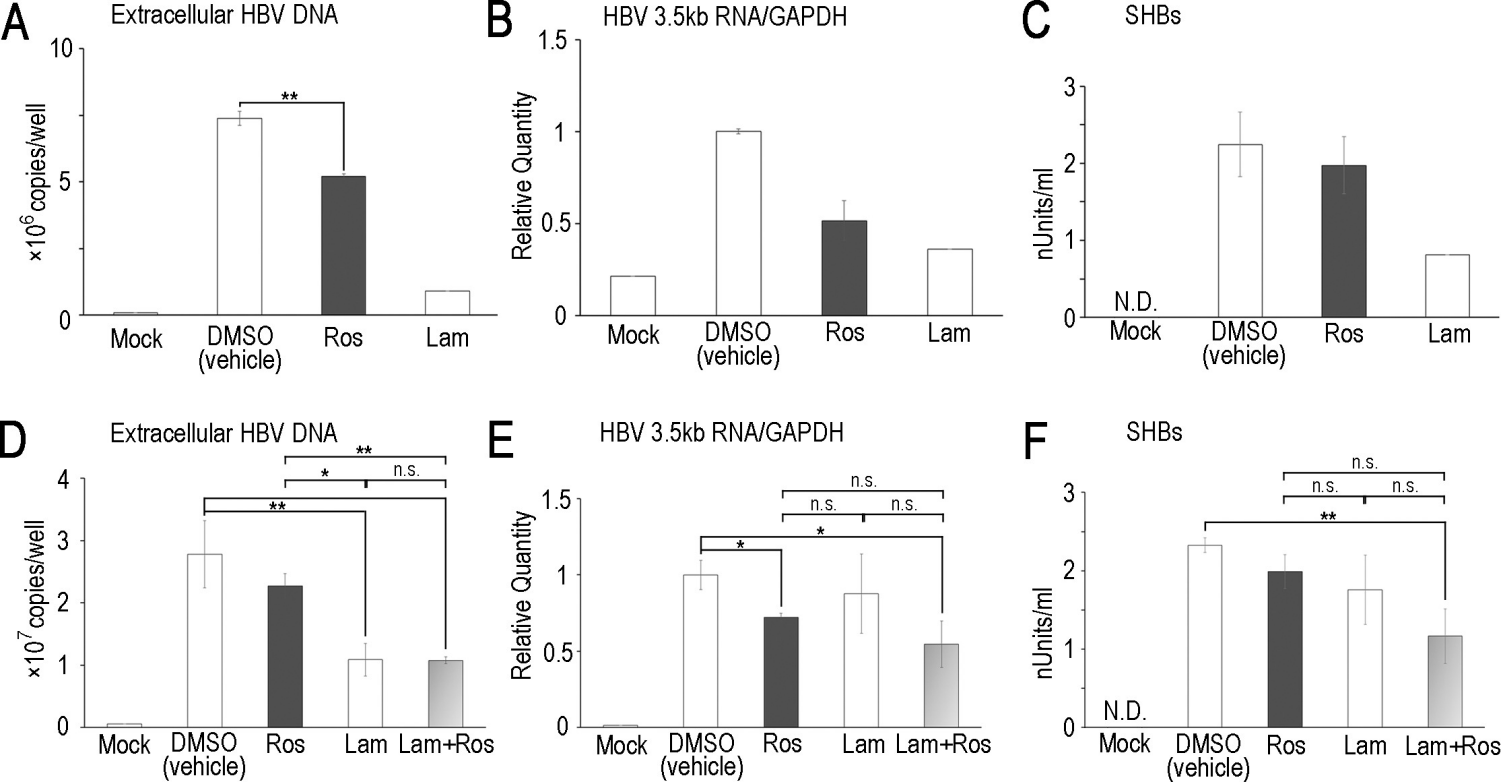
Rosmarinic acid suppresses HBV replication in HBV-infected primary human hepatocytes. (A-C) PXB-cells were infected with HBV, and treated with 30 μM rosmarinic acid or 500 nM lamivudine. Seven to 12 days post-infection, extracellular HBV DNA was quantified by qPCR, intracellular HBV 3.5 kb RNA was quantified by RT-qPCR, and SHBs were measured by ELISA. (D-F) PXB-cells were infected with HBV, and were treated with 30 μM rosmarinic acid and/or 20 nM lamivudine. Extracellular HBV DNA, intracellular HBV 3.5-kb RNA, and SHBs were measured as in (A-C). Data are from one representative of at least two independent experiments; the means and S.D. of duplicate or triplicate experiments are shown (* *p* < 0.05, ** *p* < 0.01). N.D.: not detected, n.s.: not significant.

**Fig 5:**
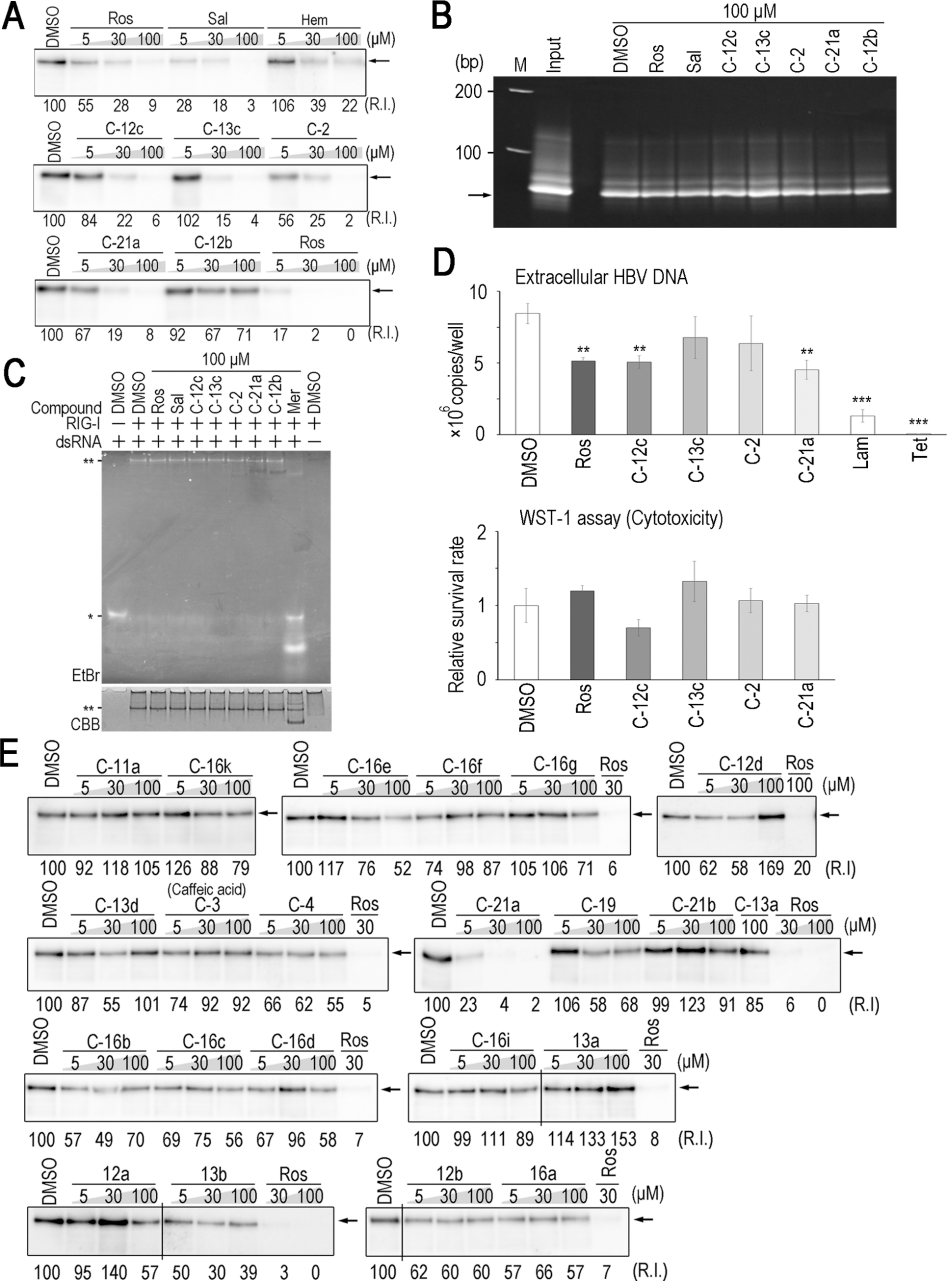
Rosmarinic acid derivatives also inhibit ε-Pol binding. (A) Western blot analysis for Pol pulled-down by ε-biotin in the presence of the indicated compounds. Arrows: 3×FLAG-Pol. (B) ssDNA-biotin was pulled-down in the presence of the indicated compounds, and detected by EtBr staining. Arrow: 71-base ssDNA. (C) dsRNA-RIG-I EMSA in the presence of the indicated compounds (100 μM). *: monomeric dsRNA, **: the dsRNA-RIG-I complex. (D) Hep38.7-Tet cells were treated with 10 μM rosmarinic acid, compounds 12c, 13c, 2, and 21a, 100 nM lamivudine, or 400 ng/ml tetracycline. Extracellular HBV DNA and cell viability were measured as in (Fig 3A and 3B). Data are from one representative of at least two independent experiments; the means and S.D. of triplicate experiments are shown (* *p* < 0.05, ** *p* < 0.01, *** *p* < 0.001). (E) A Western blot analysis for Pol pulled-down by ε-biotin in the presence of the indicated compounds. Arrows: 3×FLAG-Pol. Spliced images are indicated with a dividing line.

Quercetin was previously reported to inhibit HBV replication in HepG2.2.15 cells [32], a HepG2-derived cell line stably expressing HBV. Thus, we focused on the anti-HBV activity of rosmarinic acid using cell lines stably expressing HBV. No obvious cytotoxicity of rosmarinic acid was observed up to 30 μM in HepG2 cells and Hep38.7-Tet cells, a tetracycline inducible HBV-expressing cell line [27] (Fig 3B and 3D). The cytotoxicity of rosmarinic acid in HepG2 cells was also evaluated (Table 1). Hep38.7-Tet cells were treated with rosmarinic acid, and the amount of HBV DNA secreted into the culture medium was quantified. The rosmarinic acid treatment significantly decreased extracellular HBV DNA with rough estimation of EC50 30 μM (Fig 3A). We then performed a combined treatment with rosmarinic acid and lamivudine. Although a low concentration of lamivudine (10 nM) exerted weak anti-HBV effects, the combined treatment enhanced inhibition (Fig 3C). PXB-cells, primary human hepatocytes isolated from an immunodeficient mouse with a humanized liver, were subjected to HBV infection and the effects of rosmarinic acid on its replication were examined. Rosmarinic acid showed no toxicity up to 100 μM (S3 Fig). Extracellular HBV DNA levels was significantly reduced and intracellular HBV RNA and extracellular HBsAg levels were slightly reduced by rosmarinic acid, suggesting that rosmarinic acid suppresses HBV replication in infected cells (Fig 4A–4C). In PXB-cells, the combined treatment with rosmarinic acid and lamivudine did not further enhance inhibition (Fig 4D–4F). Collectively, these results suggest that the rosmarinic acid treatment suppresses HBV replication in infected cells. Of note, HBV-infected PXB-cells were also treated with quercetin, and its inhibitory activity on HBV replication was confirmed (S4A–S4C Fig). We analyzed the rosmarinic acid derivatives listed in Table 2. Salvianolic acid A, Compound 2, 12c, 13c, and 21a [28] exhibited inhibitory activities against ε-Pol binding in dose-dependent manners (Fig 5A). Similar to rosmarinic acid, these 5 derivatives did not affect biotin-avidin binding, dsRNA-RIG-I binding, or RIG-I helicase activity (Fig 5B, 5C and S2B Fig). Furthermore, these derivatives inhibited HBV DNA production at similar levels to that of rosmarinic acid with low cytotoxicity (Fig 5D and Table 1). On the other hand, other derivatives did not exhibit inhibitory activities (Fig 5E).

**Table 2:** Rosmarinic acid derivatives.

Compound 2	(*R*)-2-(3,4-Dihydroxyphenyl)-1-carbamoylethyl 3-(3,4-dihydroxyphenyl)acrylate
Compound 3	Caffeic acid
Compound 4	Methyl caffeate
Compound 11a	2-Phenylethyl cinnamate
Compound 12a	2-(3,4-Dihydroxyphenyl)ethyl cinnamate
Compound 12b	2-(3,4-Dimethoxyphenyl)ethyl caffeate
Compound 12c	2-(3,4-Dihydroxyphenyl)ethyl caffeate
Compound 12d	Phenylethyl caffeate
Compound 13a	Phenylethyl 3-phenylpropanoate
Compound 13b	2-(3,4-Dihydroxyphenyl)ethyl 3-phenylpropanoate
Compound 13c	2-(3,4-Dihydroxyphenyl)ethyl 3-(3,4-dihydroxyphenyl)propanoate
Compound 13d	2-(3,4-Dimethoxyphenyl)ethyl 3-phenylpropanoate
Compound 16a	2-​propenyl caffeate
Compound 16b	Propyl caffeate
Compound 16c	Butyl caffeate
Compound 16d	Pentyl caffeate
Compound 16e	Hexyl caffeate
Compound 16f	Heptyl caffeate
Compound 16g	Nonyl caffeate
Compound 16i	5-Hydroxypentyl caffeate
Compound 16k	Hydroxyhexy caffeate
Compound 19	1-(3’,4’-Dihydroxyphenyl)non-1-en-3-one
Compound 21a	*N*-caffeoyldopamine
Compound 21b	Pentyl 1-(3’,4’-dihydroxyphenyl)propen amide

## Discussion

In the present study, we established a pull-down assay to screen for inhibitors of HBV ε-Pol binding. 3×FLAG-tagged full-length Pol was expressed in human cell lines, and cell lysates were used in the assay. It was not necessary to include an exogenous chaperone protein to detect the ε-Pol interaction. The ε-Pol interaction was detected in liver-derived (HepG2) and non-liver (HEK-293T) cells. Due to its high transfection efficiency, we used HEK-293T cells for screening. In order to test a larger number of chemicals, we mixed 10 compounds in primary screening. Although mixed chemicals may exhibit unexpected interference from each other, we identified 5 out of 3,965 chemicals with inhibitory activities. Rosmarinic acid and quercetin appeared to specifically inhibit the ε-Pol interaction.

In Fig 4E, PXB cells treated with rosmarinic acid had significantly lower intracellular 3.5kb HBV RNA levels than DMSO-treated cells. Also, combined treatment with rosmarinic acid and lamivudine had lower 3.5kb HBV RNA level. This observation was unexpected if rosmarinic acid solely targets ε-Pol interaction. However, HBV progenies produced from PXB cells are known to have the potential to re-infect the cells. When rosmarinic acid specifically blocks the epsilon-polymerase binding, the treatment is estimated to reduce the production level of virus progenies and the rate of the secondary infection to PXB cells as well as to inhibit cccDNA synthesis from the pre-genome 3.5 kb HBV RNA. In such a context, we assume that HBV RNA levels in PXB cells were decreased by the treatment of rosmarinic acid.

**Fig 6:**
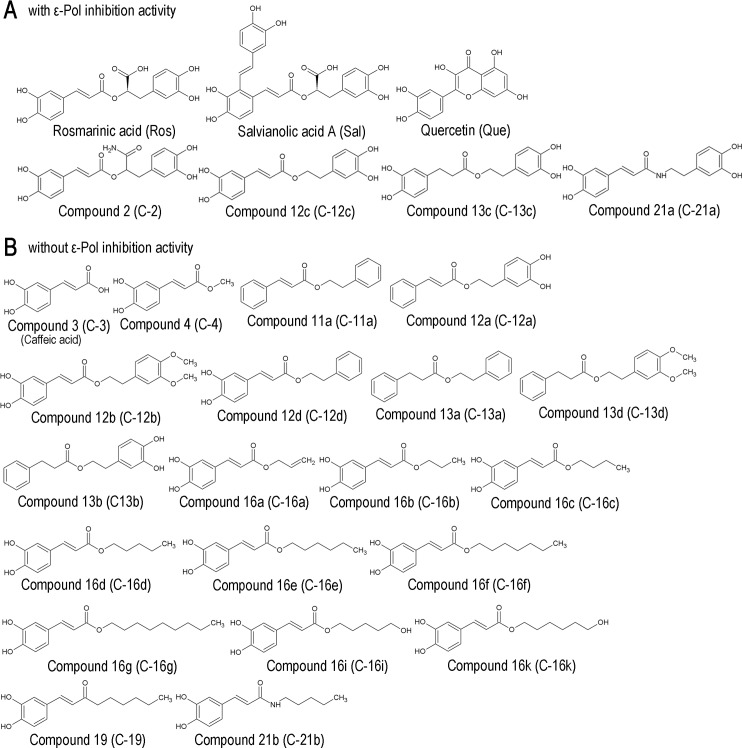
Chemical structures. Chemical structures of compounds with ε-Pol inhibitory activity (A), and without ε-Pol inhibitory activity (B).

Rosmarinic acid and quercetin are structurally related and both have two dihydoxybenzene moieties (Fig 6A). We tested the derivatives of rosmarinic acid and found that some did not exhibit inhibitory activities against the ε-Pol interaction. The examination with rosmarinic acid and its derivatives revealed a relationship between its chemical structure and ε-Pol inhibition (Figs 5 and 6). Compounds with inhibitory activities commonly had two dihydoxybenzene moieties (Fig 6A); however, caffeic acid and its derivatives (C-4, -16a-k, -19, and -21b) did not exhibit inhibitory activity. Comparisons between C-12c and C-11a revealed that the two phenolic hydroxyl groups in the catechol structure were critical and the presence of 4 complete hydroxyl groups was required for inhibitory activity (compare C-12c with C-12a and C12d). Similarly, modifications to the hydroxyl groups into–OCH_3_ groups (hydroxyl group masked with CH_3_) resulted in the loss of inhibitory activity (compare C-12c with C-12b). On the other hand, the linker structure connecting the two catechol structures appeared to be flexible (compare C-12, C-2, C-13c, and C-21a). These results suggest that the 4 hydroxyl groups in the two catechol structures at both side chain ends are critical “active domains” for recognizing Pol. Whereas the linker structure was less critical for the specific interaction but the linker may determine critical distance between the two active domains.

RIG-I is a viral RNA sensor for eliciting several antiviral responses [33, 34]. Previous studies reported that innate immune signaling is important for anti-HBV responses [26, 30]. ISG20 was shown to inhibit HBV replication in nuclease-dependent and -independent manners [31, 35]. Since rosmarinic acid and quercetin inhibit ε-Pol binding without affecting dsRNA-RIG-I binding, RIG-I helicase activity, or ε-ISG20 binding (Figs 1D, 2B and 2C and S2A Fig), they presumably do not interfere with the host antiviral immune response.

Despite ε-Pol binding being strongly abolished by the rosmarinic acid treatment *in vitro*, the suppression of HBV replication in cells by rosmarinic acid was less efficient than the nucleotide analogue, lamivudine. One of the reasons for insufficient inhibition is permeability. Another potential reason is chemical stability in cells. These limitations may be overcome in future studies. Rosmarinic acid is a natural compound (abundant in *Lamiaceae* herbs including spearmint, sage, peppermint, and perilla) and is utilized as a dietary supplement as well as Chinese herbal medicine. To date, no detrimental effects on humans have been reported, suggesting lower toxicity *in vivo*. Therefore, if we optimize drug administration, we may be able to evaluate the anti-HBV effects of rosmarinic acid *in vivo*.

## Supporting information

S1 Fig Pull-down assay-based screening for the search for novel ε-Pol binding inhibitors. (A) A Western blot analysis for Pol pulled-down by ε-biotin in the presence of a chemical mix containing 10 kinds of compounds. The final concentration of each compound was 30 μM. A total of 3,965 compounds were screened. (B) Candidate mixes were divided into single compounds, and analyzed as in (A). The final concentration of the compounds was 30 μM. Arrows: 3×FLAG-Pol.
(TIF)

S2 Fig Effects of compounds on RIG-I helicase activity. The RIG-I helicase assay was conducted in the presence of the indicated compounds. #: monomeric ssRNA, ##: annealed dsRNA, (n.s.): non-specific band.
(TIF)

S3 Fig Cytotoxicity of rosmarinic acid in HBV-infected primary human hepatocytes. PXB cells were infected with HBV, and treated with Rosmarinic acid at indicated concentrations. On day 12, cells were subjected to WST-1 cell proliferation assay. Data are from one representative of at least two independent experiments; means and S.D. of duplicate experiments are shown.
(TIF)

S4 Fig Quercetin suppresses HBV replication in HBV-infected primary human hepatocytes. PXB cells were infected with HBV, and treated with 30 μM Quercetin. Extracellular HBV DNA, intracellular HBV 3.5 kb RNA, and SHBs were measured as in Fig 4A–4C. Data are from one representative of at least three independent experiments; means and S.D. of duplicate experiments are shown (* p < 0.05).
(TIF)

S5 Fig Original gels and Western blots. Uncropped and unadjusted gels and Western blots.
(TIF)
