# Supplementary figures and images for: Rosmarinic acid is a novel inhibitor for Hepatitis B virus replication targeting viral epsilon RNA-polymerase interaction
Source: PLoS One. 2018 May 21;13(5):e0197664. doi: 10.1371/journal.pone.0197664 (PMC5962091; doi:10.1371/journal.pone.0197664)

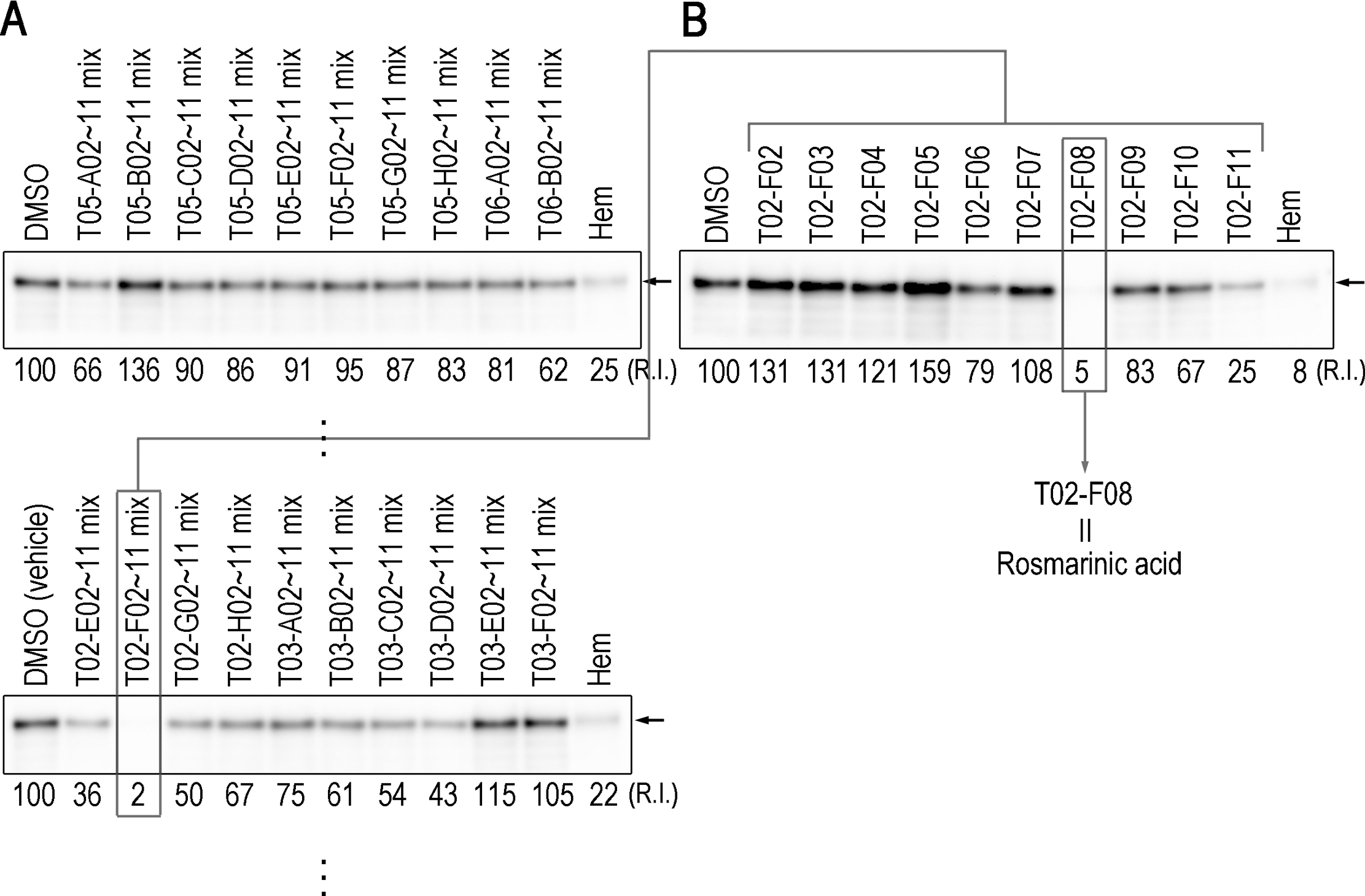

Supplement: S1 Fig — (A) A Western blot analysis for Pol pulled-down by ε-biotin in the presence of a chemical mix containing 10 kinds of compounds. The final concentration of each compound was 30 μM. A total of 3,965 compounds were screened. (B) Candidate mixes were divided into single compounds, and analyzed as in (A). The final concentration of the compounds was 30 μM. Arrows: 3×FLAG-Pol. (TIF) [file pone.0197664.s001.tif]

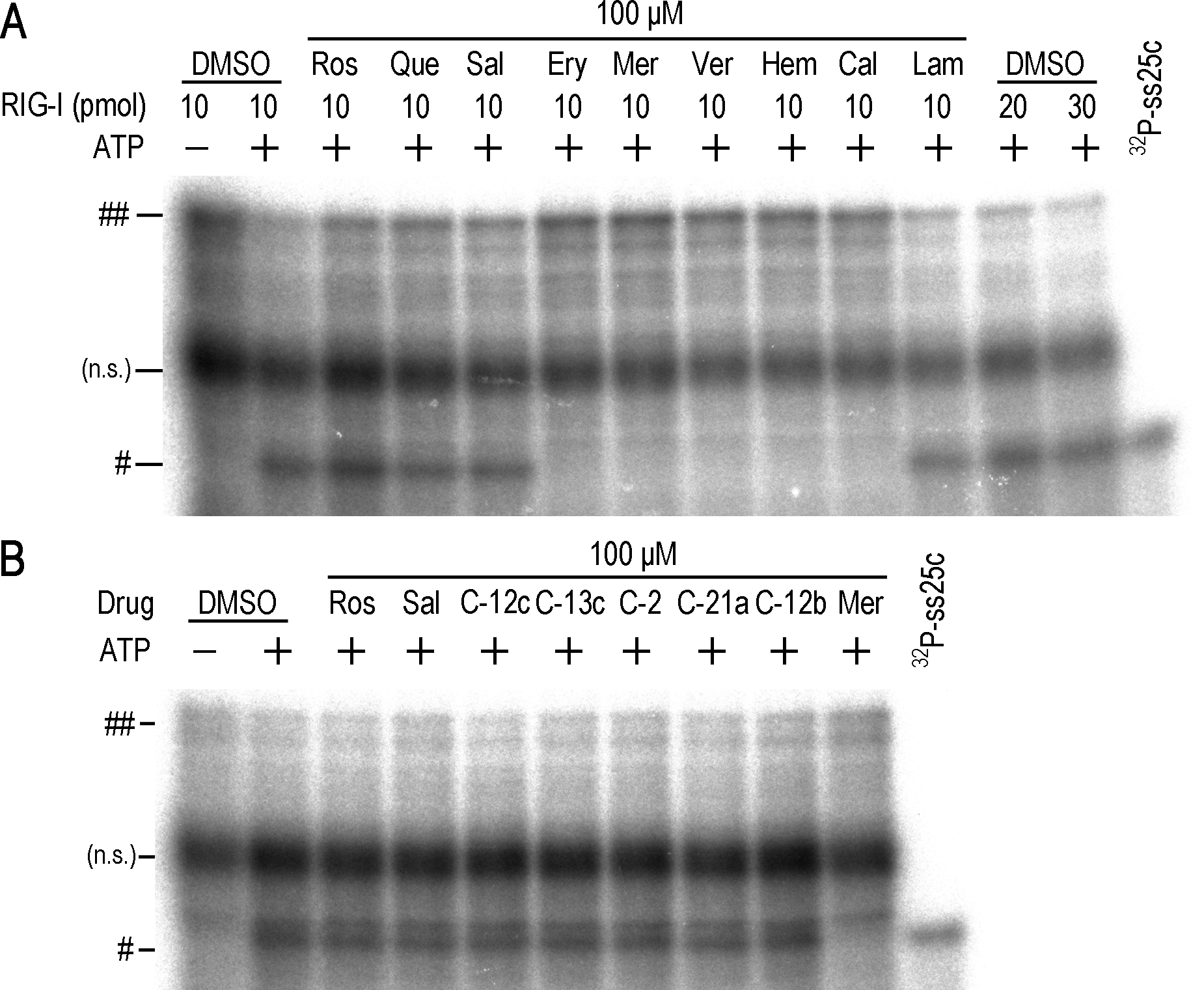

Supplement: S2 Fig — The RIG-I helicase assay was conducted in the presence of the indicated compounds. #: monomeric ssRNA, ##: annealed dsRNA, (n.s.): non-specific band. (TIF) [file pone.0197664.s002.tif]

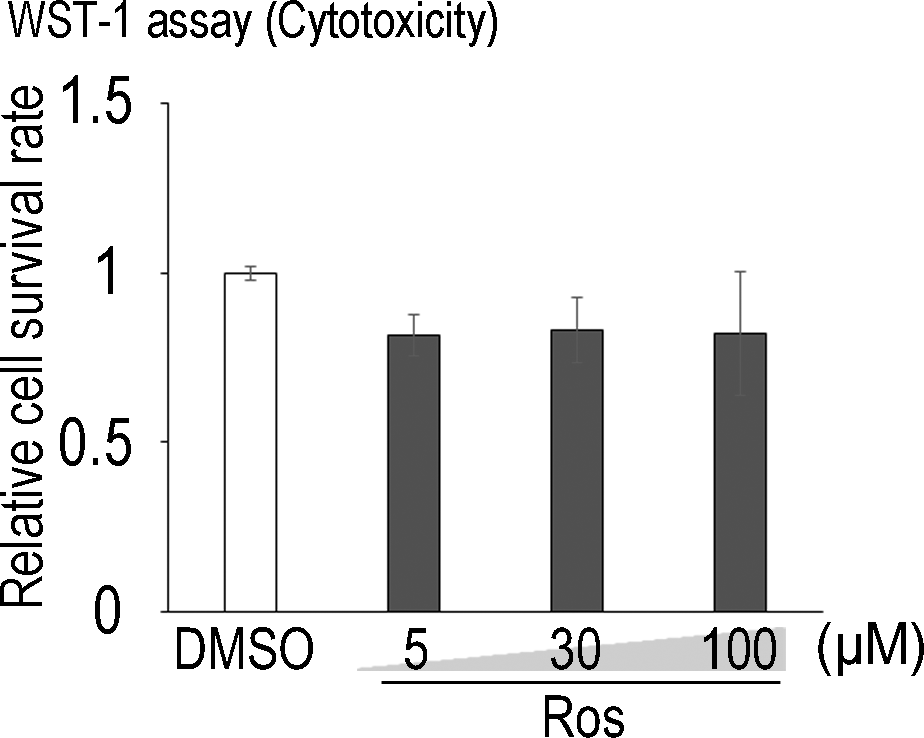

Supplement: S3 Fig — PXB cells were infected with HBV, and treated with Rosmarinic acid at indicated concentrations. On day 12, cells were subjected to WST-1 cell proliferation assay. Data are from one representative of at least two independent experiments; means and S.D. of duplicate experiments are shown. (TIF) [file pone.0197664.s003.tif]

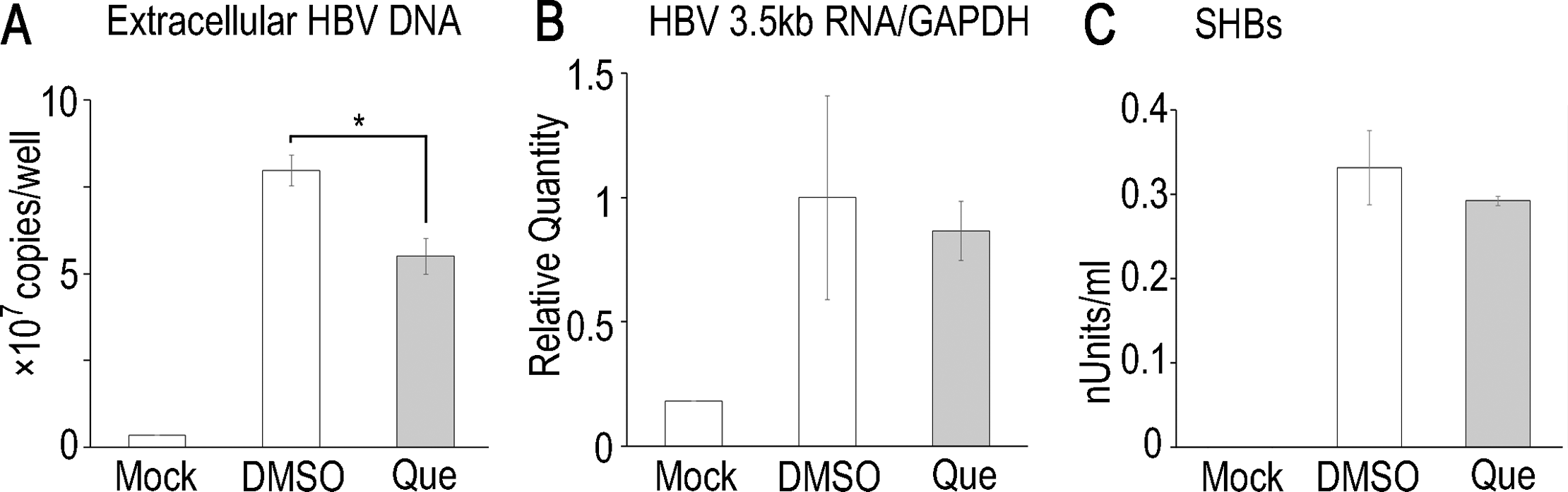

Supplement: S4 Fig — PXB cells were infected with HBV, and treated with 30 μM Quercetin. Extracellular HBV DNA, intracellular HBV 3.5 kb RNA, and SHBs were measured as in Fig 4A–4C. Data are from one representative of at least three independent experiments; means and S.D. of duplicate experiments are shown (* p < 0.05). (TIF) [file pone.0197664.s004.tif]

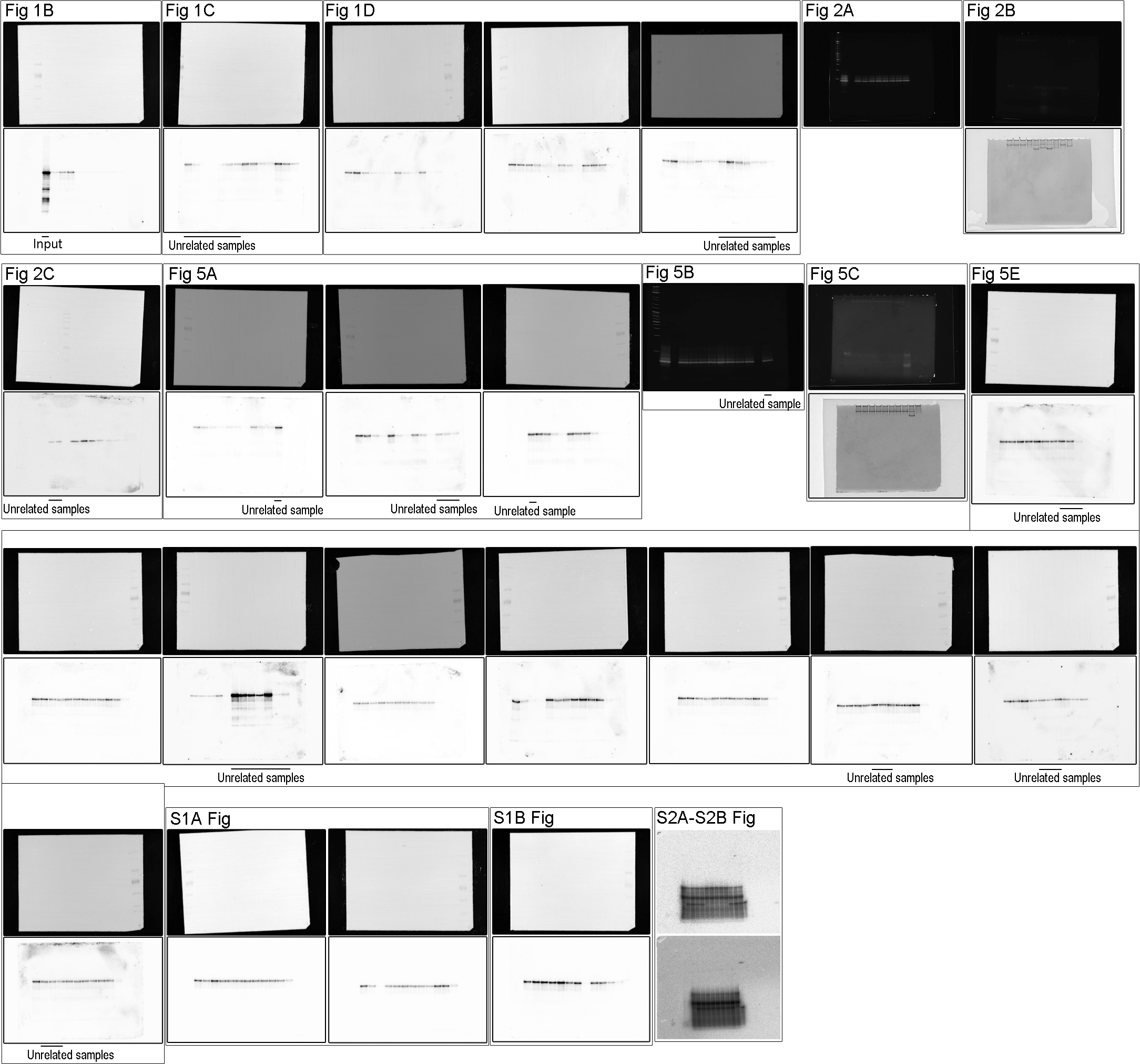

Supplement: S5 Fig — Uncropped and unadjusted gels and Western blots. (TIF) [file pone.0197664.s005.tif]
